# Preliminary Electroencephalography-Based Assessment of Anxiety Using Machine Learning: A Pilot Study

**DOI:** 10.3390/brainsci15060571

**Published:** 2025-05-26

**Authors:** Katarzyna Mróz, Kamil Jonak

**Affiliations:** Faculty of Mathematics and Information Technology, Lublin University of Technology, 20-618 Lublin, Poland; k.jonak@pollub.pl

**Keywords:** electroencephalography, brain imaging, brain–computer interface, EEG analysis, neural networks, machine learning

## Abstract

**Background**: Recent advancements in machine learning (ML) have significantly influenced the analysis of brain signals, particularly electroencephalography (EEG), enhancing the detection of complex neural patterns. ML enables large-scale data processing, offering novel opportunities for diagnosing and treating mental disorders. However, challenges such as data variability, noise, and model interpretability remain significant. This study reviews the current limitations of EEG-based anxiety detection and explores the potential of advanced AI models, including transformers and VAE-D2GAN, to improve diagnostic accuracy and real-time monitoring. **Methods**: The paper presents the application of ML algorithms, with a focus on convolutional neural networks (CNN) and recurrent neural networks (RNN), in identifying biomarkers of anxiety disorders and predicting therapy responses. Additionally, it discusses the role of brain–computer interfaces (BCIs) in assisting individuals with disabilities by enabling device control through brain activity. **Results**: Experimental EEG research on BCI applications was conducted, focusing on motor imagery-based brain activity. Findings indicate that successive training sessions improve signal classification accuracy, emphasizing the need for personalized and adaptive EEG analysis methods. Challenges in BCI usability and technological constraints in EEG processing are also addressed. **Conclusions**: By integrating ML with EEG analysis, this study highlights the potential for future healthcare applications, including neurorehabilitation, anxiety disorder therapy, and predictive clinical models. Future research should focus on optimizing ML algorithms, enhancing personalization, and addressing ethical concerns related to patient privacy.

## 1. Introduction

Understanding the functioning of the human brain has long been one of the major scientific challenges [1,2,3,4,5,6]. The brain consists of millions of neurons that play a key role in controlling bodily behavior in response to both internal and external stimuli. Neurons transmit information between the central and peripheral nervous systems via axons, enabling the regulation and coordination of motor and sensory states, such as eye and lip movements, as well as processes related to attention and memory. Emotional responses are regulated by complex neural networks that integrate different brain regions [7,8,9].

It is important to distinguish between anxiety as a clinical disorder and anxiety as a situational emotional reaction, as these forms may involve different neurophysiological mechanisms and generate distinct EEG patterns. This distinction is especially relevant in studies utilizing non-clinical or subclinical samples, where context-induced emotional states may reflect transient rather than chronic alterations in brain activity [7,8,9,10,11].

Despite recent advances in electroencephalography (EEG) and machine learning (ML), significant challenges remain in optimizing model accuracy, interpretability, and generalizability when working with EEG recordings. Traditional signal-processing methods often struggle with high-dimensional datasets (hundreds of channels and thousands of time points) and noisy EEG data, where “noise” may include biological artifacts (e.g., eye blinks, muscle contractions), environmental interferences (e.g., line noise), and low signal-to-noise ratios in critical frequency bands. This study is explicitly positioned as a pilot project due to the extremely small sample size (N = 3), which inherently limits the generalizability of the findings. While this limitation prevents meaningful inferential statistics, the pilot design provides valuable exploratory insights and methodological foundations for future, larger-scale studies [7,8,9,10,11].

Although previous reviews have focused on EEG-based anxiety detection, this review uniquely explores how emerging AI and machine learning (ML) techniques are identifying novel, quantifiable EEG biomarkers of anxiety that were previously undetectable using conventional methods. Recent studies have also proposed various frameworks for classifying anxiety levels using EEG signals and machine learning algorithms [3,12,13,14]. Muhammad and Al-Ahmadi [12] developed a classification system targeting human state anxiety in exposure therapy contexts, while Aldayel and Al-Nafjan [13] provided a comprehensive exploration of machine learning approaches for EEG-based anxiety detection. Rajendran et al. [3] analyzed EEG signals to evaluate examination stress and test anxiety among college students, and Sakib et al. [14] designed a wireless EEG-based screening model for young adults experiencing anxiety. In contrast to these studies, our work focuses on a pilot investigation using a motor imagery (MI) brain–computer interface (BCI) paradigm conducted during naturally occurring academic stress. This approach emphasizes the validation of EEG signal acquisition and machine learning pipelines within a controlled but ecologically valid context, laying the groundwork for future large-scale studies explicitly targeting anxiety-state classification [3,12,13,14,15].

This study builds upon a previous investigation conducted by Katarzyna Mróz et al. (2022), where data were collected to analyze brain–computer interface (BCI) systems for motor imagery paradigms in the context of everyday life applications [11]. In this paper, we expand on the previous findings by shifting the focus toward the user experience of individuals who participated in the study, particularly in relation to their perception of the BCI system’s applicability. While the earlier study primarily explored the use of BCI in motor imagery paradigms, this research introduces new insights gained from interviews with patients, focusing on how BCI systems could be applied to individuals with various mental health disorders. The aim of this pilot study is to critically assess the current state of EEG-based anxiety detection, highlight the limitations of traditional methods, and explore the potential of advanced machine learning techniques to improve diagnostic precision and real-time monitoring in the context of anxiety disorders. This preliminary study provides foundational insights and sets the stage for more extensive investigations [7,8,9,10].

A fundamental aspect of anxiety disorders is altered attentional control, which affects how individuals with anxiety process and focus their attention. These modifications in attentional control are associated with specific changes in brain activity, measurable through electroencephalography (EEG). EEG techniques provide valuable insights into alterations in brain function related to anxiety disorders [10,16,17,18,19,20,21,22,23]. Event-related EEG markers, such as error-related negativity (ERN) and correct-response negativity (CRN), are considered reflections of response monitoring processes and attentional control pathology in anxiety [7,8,9,10,11].

Recent research has increasingly focused on the automatic detection of anxiety using physiological signals, such as EEG, galvanic skin response (GSR), and heart rate variability. These signals offer a reliable and non-invasive means of observing changes in mental states, enabling their application in anxiety detection across various contexts. Consequently, artificial intelligence and machine learning techniques have been employed to develop systems aimed at improving the accuracy of anxiety identification, facilitating early intervention and supporting individuals affected by anxiety disorders [24,25,26].

Machine learning encompasses a range of algorithms and techniques that allow computers to identify patterns within complex datasets without explicit programming. EEG, as a method for recording brain electrical activity, generates vast amounts of data that require advanced processing and analysis methods. ML techniques not only enable the precise extraction of hidden patterns from EEG data but also support the advancement of personalized medicine in psychiatry, allowing for therapy response prediction and monitoring disorder progression [7,8,9,10,11,27,28,29,30].

Although machine learning is a relatively new tool in neurobiology, its application in EEG data analysis opens new diagnostic and therapeutic perspectives. This paper provides a comprehensive review of the potential benefits of machine learning in the analysis of brain neural networks, with a particular focus on anxiety disorders, and discusses future directions for research in this field. This paper addresses these challenges by exploring innovative methods for analyzing electroencephalographic (EEG) signals in the context of anxiety disorders, with a specific focus on the use of machine learning (ML) techniques for identifying key brain activity patterns [2,10,19,20,21,22,23,31].

This review focuses on the application of machine learning methods to the analysis of brain neural networks. The aim of this study is to provide a comprehensive review of current research on the use of ML in the analysis of electroencephalographic (EEG) signals in the context of anxiety disorders. Special attention is given to identifying specific brain activity patterns that may serve as diagnostic biomarkers for anxiety. The paper also discusses the potential benefits of integrating ML techniques with EEG analysis and outlines future research directions in this field.

## 2. Materials and Methods

### 2.1. Participants

**Figure 1:**
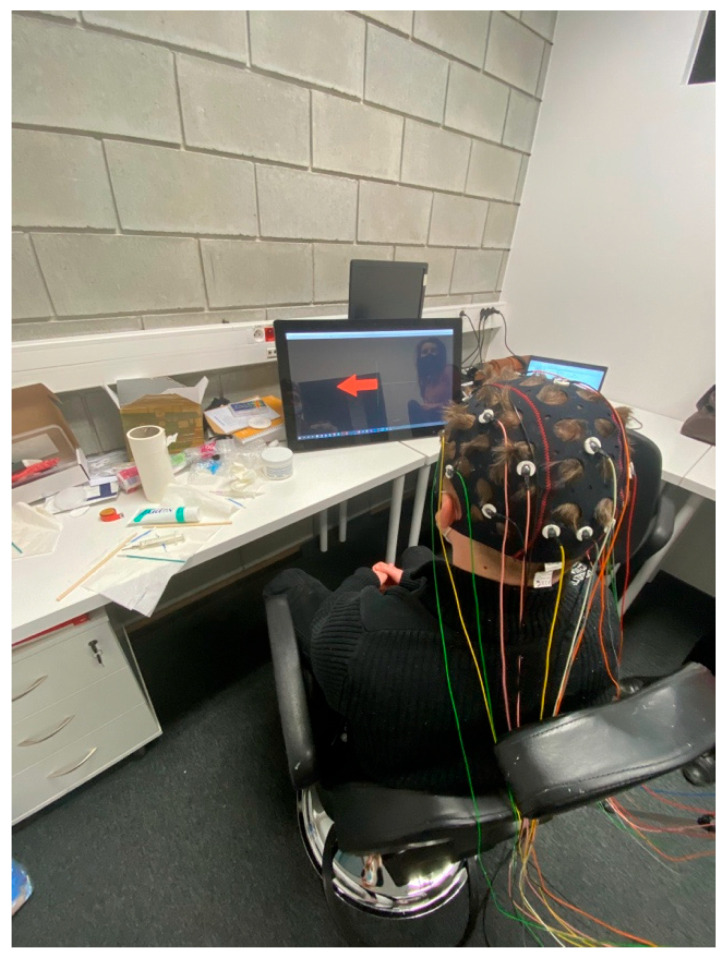
The study participant during the research procedure.

A small group of 3 participants was selected to take part in this initial phase of the study. These individuals, all aged 23, were chosen to assess the feasibility and effectiveness of the proposed method in a controlled setting. What distinguishes our study is the focus on a naturally induced anxiety context—our participants were engineering students experiencing significant academic stress during final exams and thesis defense preparation. This population represents a real-world model of acute, situational anxiety, rather than a clinical, chronic sample. While the sample size is limited, the results provide valuable insights into the potential applications of brain–computer interfaces (BCI) for motor imagery paradigms. These findings will inform future studies with larger, more diverse groups. None of the participants were taking medication, had any mental disorders, or suffered from epilepsy. Before the experiment, each participant was informed about the laboratory regulations. Additionally, detailed information about the study’s purpose and procedure was provided. Participants also signed a consent form to take part in the study. The research was approved by the Ethics Committee of Lublin University of Technology. Participants were asked to avoid making vigorous head movements and to maintain a stable body position. A specially designed chair was used to minimize unwanted movements during the experiment. The room where the study was conducted was dimly lit, which helped prevent visual distractions and allowed participants to fully focus their visual attention on the monitor screen. The dark environment contributed to better tracking results compared to a brightly lit room. Figure 1 illustrates the correct posture of a participant during the experiment.

### 2.2. Research Procedure

**Figure 2:**
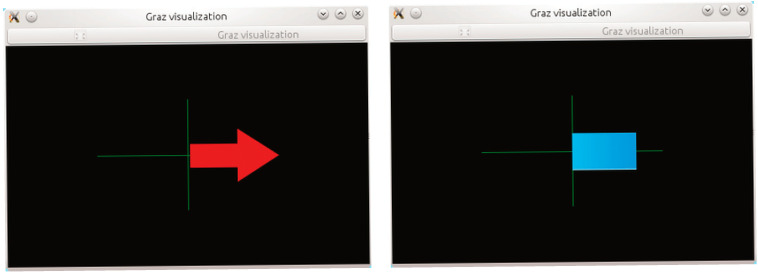
Motor imagery scenario in OpenViBE [32].

The research procedure, during which electroencephalographic (EEG) studies were conducted, required the selection of an appropriate research subject. An interface was necessary to collect the required information along with the events occurring during the experiment. Figure 2 presents its visualization. During the study, each participant was presented with an interface consisting of a board with an arrow. During the presentation, arrows pointing either to the right or to the left were displayed randomly. Depending on the stage of the study, the instructions and requirements for the participant varied. In one stage, the participant was asked to focus on the computer screen and imagine flexing either the right or left hand, depending on the direction of the arrow displayed on the monitor. In this case, the participants engaged in the process of motor imagery, which involves the mental execution of movement without actual muscle activation. Motor imagery requires the conscious activation of brain areas responsible for planning and executing movement, making it a crucial element in neurorehabilitation. In another stage, participants were required to physically perform the flexion of specific hands. This allowed for a comparison of brain activity during actual movement execution and its mental imagery. The analysis of the obtained EEG data enabled the evaluation of the effectiveness of motor imagery in activating the relevant brain regions and its potential application in the rehabilitation of neurological patients, as well as in anxiety disorder therapy by modulating neuronal activity related to movement control and emotional regulation. In addition to these stages, the study added ecological validity to the research by recording neural responses during a period of naturally elevated stress. This provided more authentic EEG correlates of anxiety than artificially induced laboratory paradigms. Furthermore, a brief resting-state EEG protocol, lasting under 4 min, was implemented to capture real-time anxiety-related neural fluctuations without requiring complex stimuli or task engagement. This approach supports the future use of EEG in fast screening applications in academic or workplace settings. Unlike many studies that rely on clinically diagnosed patients, our work demonstrates that subclinical, context-induced anxiety can be reliably captured through EEG patterns and classified using machine learning techniques. This supports the hypothesis that EEG biomarkers may not only aid in diagnosing disorders but also in identifying transient or emerging emotional dysregulation.

**Figure 3:**
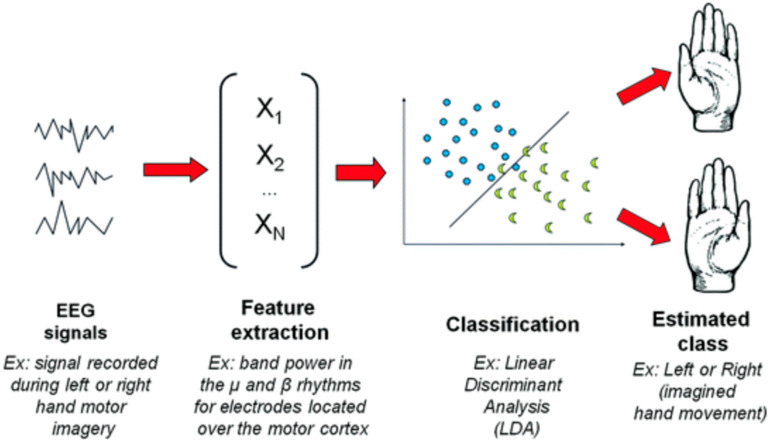
A classical EEG signal processing pipeline for BCI, in the context of a motor imagery-based BCI [33].

Figure 3 illustrates the classical EEG signal processing pipeline used in Brain–Computer Interface (BCI) systems, specifically in the context of motor imagery-based BCI, where imagined movements are recognized from EEG signals. The figure highlights the following key stages involved in processing EEG signals for movement intention recognition:

1. EEG Signal Acquisition: The initial stage involves recording brain activity via EEG electrodes placed on the participant’s scalp. The EEG signal captures brain waves associated with motor imagery tasks, such as imagined movements of the limbs.
2. Preprocessing and Filtering: Raw EEG data undergoes preprocessing to remove noise and artifacts, improving the signal quality. This step is critical for ensuring that only the relevant brain activity related to motor imagery is retained for further analysis.
3. In this study, preprocessing involved the following critical steps to enhance EEG signal quality:
  - A band-pass filter (0.1–40 Hz) was applied to eliminate slow drifts and high-frequency noise.
  - A notch filter at 50 Hz was used to remove power line interference.
  - Artifact reduction was performed using spatial Laplacian filtering, which minimized muscle and ocular artifacts by enhancing local brain activity signals.
4. Feature Extraction: In this stage, important features related to brain activity are extracted from the preprocessed EEG signals. These features capture the neural signatures associated with imagined movements and are used as inputs for the classification model.
5. Classification: The extracted features are then fed into a classification algorithm (LDA), which classifies the brain activity into different categories, such as “left-hand movement” or “right-hand movement”, based on the participant’s motor imagery.
6. Output and Feedback: Finally, the BCI system generates a classification result that can be used to control external devices or provide feedback to the user. This feedback can guide the user in performing motor tasks or interacting with the system.

### 2.3. Research Scenario

To achieve the research objective, the “Motor Imagery” scenario available in the OpenViBE 3.2.0 software was utilized. This scenario consists of six stages: signal monitoring, data acquisition, interface training, classifier training, online testing, and replay. Each participant underwent two experimental trials. In the first trial, participants engaged in a training session where they squeezed rubber balls using the muscular activity of both their right and left hands. The second trial, however, did not involve the use of the balls; instead, participants were instructed to mentally imagine the movement of their hands. This phase was based on the motor imagery paradigm.

For data classification, the “Motor Imagery” scenario employed a Linear Discriminant Analysis (LDA) classifier in combination with a k-fold cross-validation algorithm. The LDA model was selected due to its simplicity, robustness, and suitability for small sample sizes. LDA is known for its low computational complexity and its ability to perform both feature reduction and classification, making it an ideal choice for EEG data where interpretability and efficiency are critical.

Given the exploratory and pilot nature of this study and the very limited dataset (N = 3), only the LDA model was utilized. In contrast to the more complex models explored in other scenarios of our study (e.g., CNN, VAE-D2GAN, Transformer), LDA was deliberately chosen due to its minimal model complexity, which helps to avoid overfitting in extremely low-data regimes. These more advanced models, although promising, are not well-suited for datasets of this size, as their training typically requires substantially larger volumes of data to yield reliable and interpretable results.

To address the risk of overfitting inherent to small-sample research, a k-fold cross-validation approach was employed (with k = 7), which allowed for maximum use of the data in training and validation cycles. This procedure was chosen to mitigate overfitting and to obtain a more reliable estimate of model performance, despite the dataset’s small size. We acknowledge the limitation that, due to the very limited number of participants, no formal generalization error metrics (e.g., learning curves, validation loss plots) could be generated in a meaningful way. These issues will be addressed in future studies with larger samples.

The LDA classifier is responsible for feature reduction, allowing for more efficient data management before classification. Meanwhile, the k-fold cross-validation algorithm ensures that the entire dataset is utilized for both model validation and training. Additionally, during the training process, this method assesses model quality to mitigate the risk of overfitting. Below is a detailed description of each stage within the “Motor Imagery” scenario [34].

**Figure 4:**
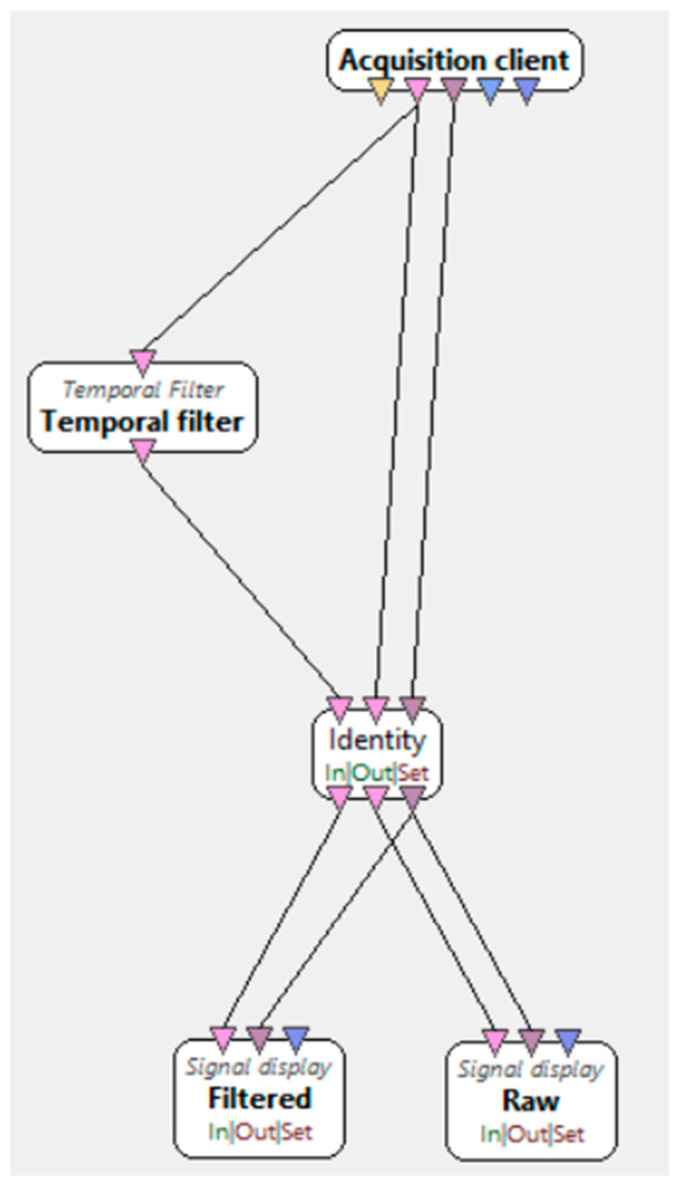
Motor imagery—signal monitoring [34].

“Motor imagery—signal monitoring”, presented in Figure 4, is used to check the quality of signals collected from the electrodes before starting the experiment. This is achieved by asking the participant to blink or close their eyes. At that moment, alpha waves become visible in the EEG Studio 1.9.7445 software, as their occurrence is associated with eye closure. Once the signal quality is confirmed to be correct, the acquisition device can proceed to the next stage.

**Figure 5:**
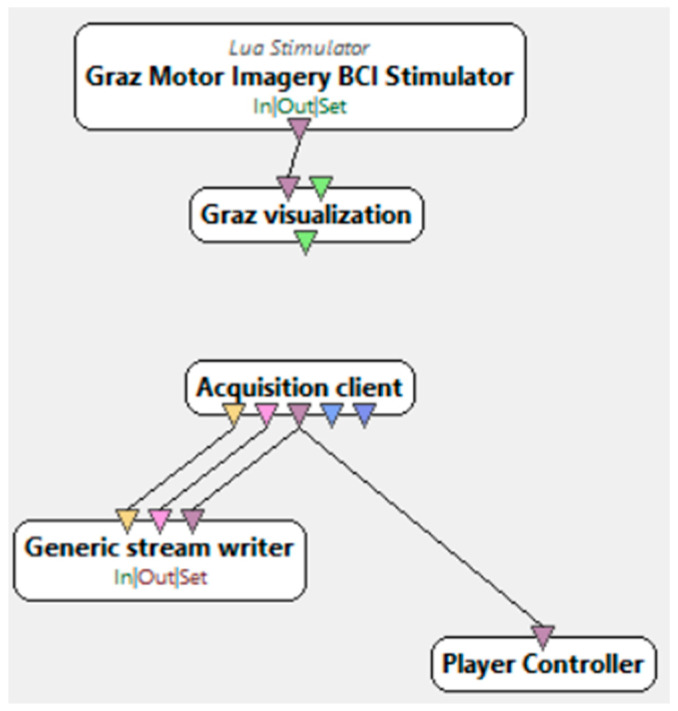
Motor imagery—acquisition [34].

“Motor imagery—acquisition”, presented in Figure 5, is the stage of data acquisition, during which training data are collected. The gathered data are used for training a classifier responsible for distinguishing between right-hand and left-hand movement. Starting this stage triggers the appearance of a black window on the computer monitor, where a sequence of instructions begins after 40 s. This sequence involves displaying a fixation cross, followed by randomly appearing arrows pointing either to the right or left. The interface user is instructed to imagine moving their right hand when a right arrow appears and their left hand when a left arrow appears. Each direction is presented 20 times, resulting in a total of 40 simulation trials.

**Figure 6:**
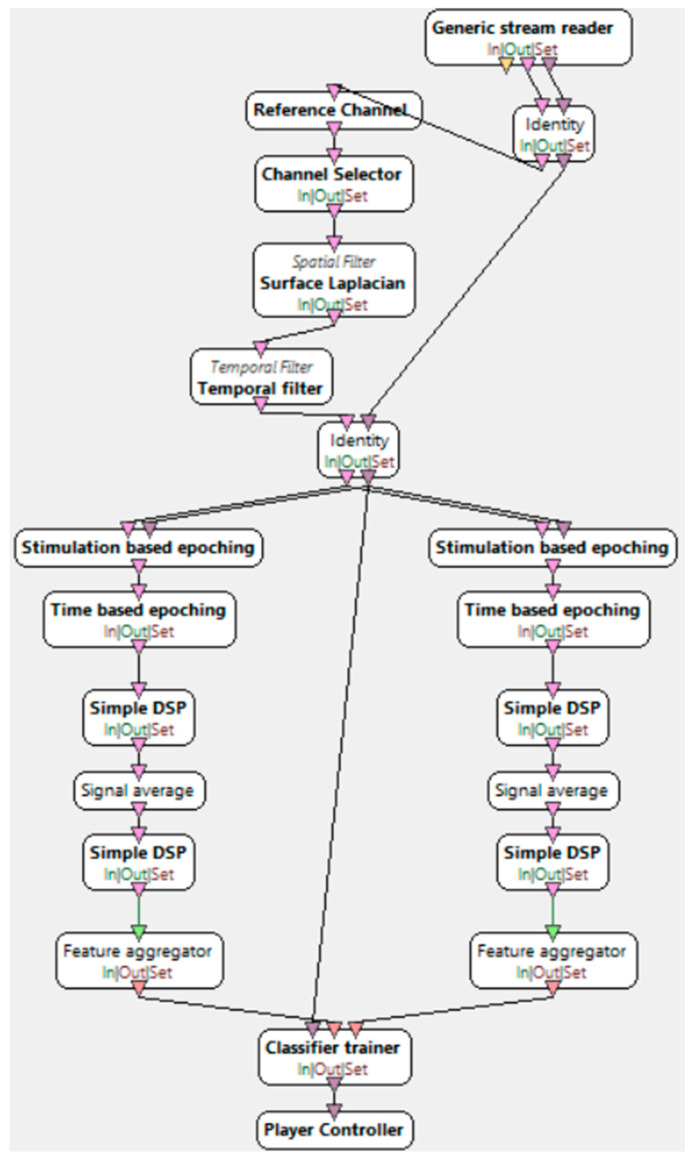
Motor imagery—classifier trainer [34].

“Motor imagery—classifier trainer”, presented in Figure 6, is the stage of training the LDA classifier based on the data obtained from the previous stage. This process involves applying the spatial filter acquired earlier before feature extraction. Feature extraction is performed by filtering the signal in the alpha/beta range. Next, the EEG epoching procedure takes place, where specific time windows are selected. Four seconds of the signal are extracted, starting half a second after the instruction is displayed to the user. The signal is divided into one-second blocks within a 16 s range. At the end of this stage, a classifier configuration file is generated, which is used in the next step—the online session.

**Figure 7:**
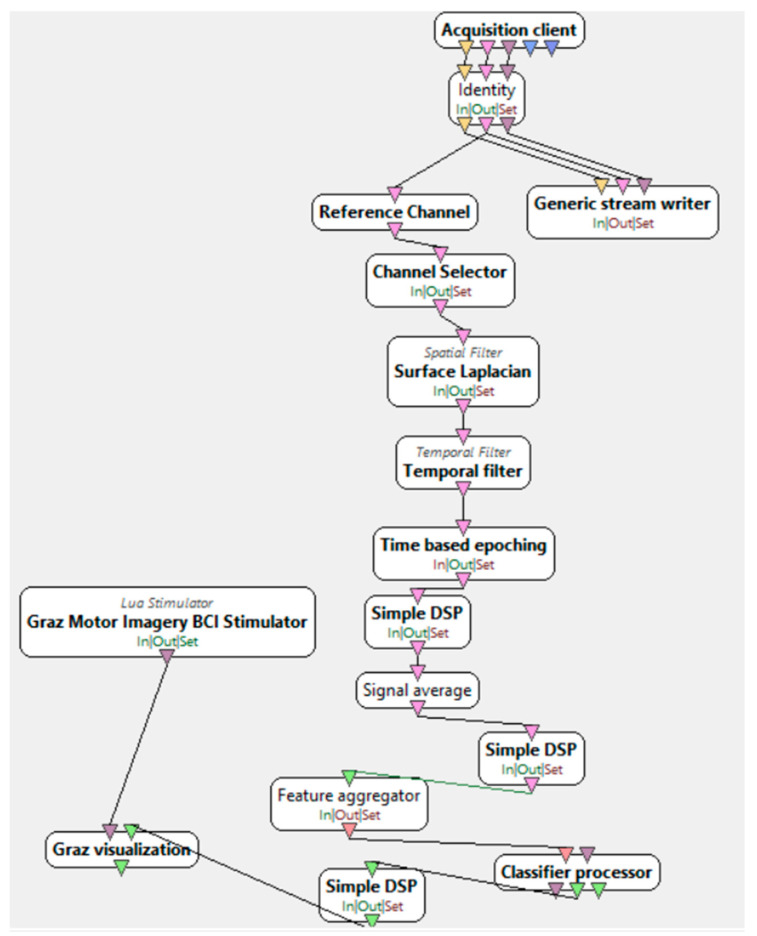
Motor imagery—online [34].

“Motor imagery—online”, as presented in Figure 7, is a stage conducted after training the classifier. It involves similar data processing procedures as in the previous stage, except for adding real-time feedback visualization. This is achieved using the trained LDA classifier from the previous stage. The expected feedback values should be negative for one class and positive for the other.

**Figure 8:**
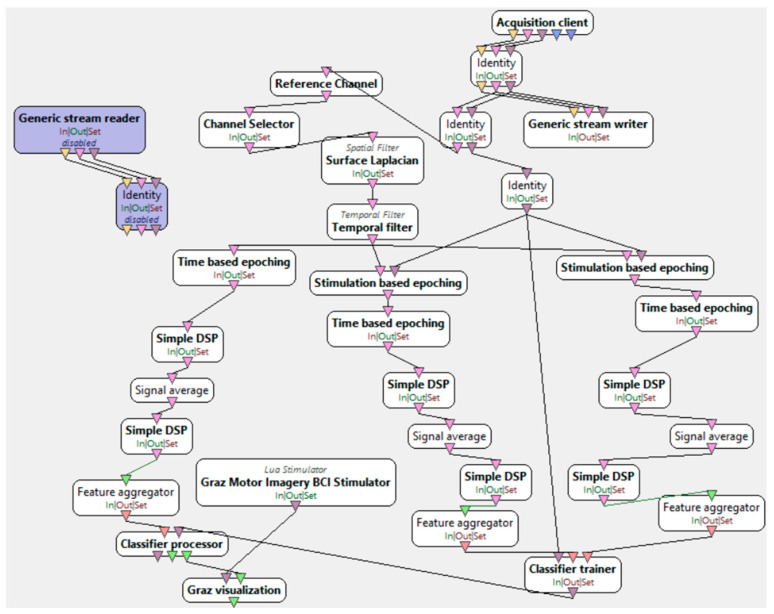
Motor imagery—online incremental [34].

“Motor imagery—online incremental”, as presented in Figure 8, is a stage conducted similarly to the previous one, except for the expectations regarding correct feedback.

**Figure 9:**
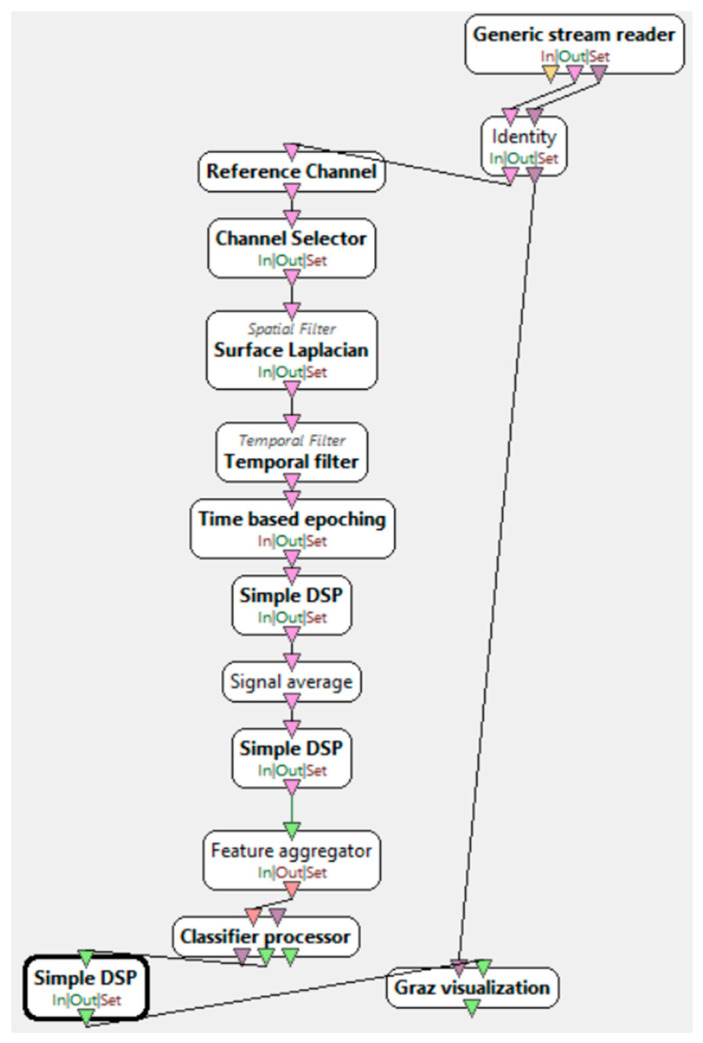
Motor imagery—replay [34].

“Motor imagery—replay”, as presented in Figure 9, is a stage based on the previous “Motor imagery—online”, allowing the playback of a recorded file during an online session at an accelerated speed. The difference between them lies in the input signal source, which comes from a file rather than from the acquisition server.

## 3. Results

The results obtained during classification are presented in several tables. The extracted data come from training the classifier using the k-fold cross-validation algorithm. The specified data were recorded during each session of individual users.

### 3.1. Classification Results

The results of evaluating the performance of the LDA classifier based on the k-fold cross-validation method are shown in Table 1. A value of k = 7 was used for the analysis, meaning that the data were divided into seven subsets, six of which were used for training and one for validation. This process made it possible to evaluate the effectiveness of the classification algorithm, which reflects the model’s level of learning during the training phase. The results obtained for the LDA classifier indicate very good learning quality. The average accuracy value obtained by k-fold cross-validation for all test participants was over 60%. The best results were achieved by the last tested BCI user, while the weakest results were recorded for the second tested participant.

The person who achieved the best results made a request at the beginning of the study to darken the laboratory room additionally, even though the room was already dark enough. This request may indicate that it helped her significantly to achieve the best results. Prior to the start of the testing procedure, the participant showed great interest in the process, asking detailed questions, which may have indicated his wholehearted approach. During the test, the person was extremely relaxed and focused. After the test, the participant admitted that she consciously sought to relax, as suggested by the researchers. The participant stressed that she had avoided excessive physical and intellectual activity to rest before the test, and she consciously did not use social media to avoid becoming emotionally stimulated.

The participant who obtained the lowest score in the study mentioned after its completion that the procedure itself was a pleasant and interesting experience. However, an important factor that could have influenced their results was their life situation—the study was conducted a day before their makeup exam, which might have caused stress and anxiety. Since the participants were students, and individuals at this stage of life often experience high levels of academic stress, it can be assumed that psychological tension may have impacted the test results. Stress can significantly affect brain function, and its influence is clearly visible in EEG recordings. High levels of anxiety and tension can lead to increased muscle tension, which in turn may interfere with the electrical signals recorded by the electrodes. Increased activity in the nervous system can also affect brain wave rhythms, leading to a higher presence of beta waves (associated with alertness and anxiety) and a decrease in alpha waves, which are linked to relaxation states.

Table 2 presents the LDA classifier’s learning outcomes obtained through k-fold cross-validation during the initial training phase, where participants physically engaged their muscle activity by alternately flexing their hands instead of using the motor imagery paradigm. The obtained results are significantly worse than those presented in Table 1, despite being derived from the same users and following the same classification procedure. The average k-fold cross-validation value exceeds 40%.

The results of the study indicate that this was the participants’ first exposure to the BCI system, which may have led to atypical neuronal responses related to adapting to the new task. The stress associated with this new experience, combined with the potential impact of anxiety states, could have influenced the reduced classification performance and lower efficiency during the initial session of the experiment. The difficulties observed in the early phase suggest that BCI systems should include extended acclimatization phases and a gradual introduction of users to the technology in order to minimize stress impact and maximize performance. Future studies should carefully analyze the mechanisms of neural adaptation, taking into account the role of emotional regulation, and develop individualized strategies that could accelerate the learning process in BCI applications. An increased number of training sessions before the actual study, particularly for young individuals suffering from anxiety or stress, could significantly improve user performance. In this context, it will be crucial to focus not only on the training during the study but also on its preparatory phases, which may constitute an essential component for improving outcomes at the beginning of the adaptation process.

The next part of the analysis concerns the results during the classifier’s learning phase regarding the predicted intentions of the user. The task of the classifier was to trigger a pulse in the form of an arrow relative to the defined stimulus, which means determining the direction of the arrow based on the hand flexion movement the participant imagines. At the end of the training, two matrices were obtained. The first one presented the cross-validation, and its results are shown in Table 3.

The results of the confusion matrix show the equivalence of the feature vector. This indicates the quality of the classifier, for example, whether the accuracy is significantly biased towards one class. For target 1, which is the imagined movement of the right hand, the predicted class values for all users were above 70%. This means that the feature vector for target 1 was correctly predicted as target 1 in 70% of the cases. This is a very high result. Similarly, for target 2, which is the imagined movement of the left hand, the predicted class values for all users were above 65%. The results were slightly lower, which may be due to the right-handedness of all the participants, affecting the ease of imagining target 1. Therefore, the trained classifier is balanced with respect to both feature vectors.

The second matrix obtained at the end of the training was the confusion matrix for the learning task. Its results are presented in Table 4.

The results of the training data matrix represent the percentage of correct arrow triggers on the monitor screen based on the motor imagery generated by the participant. The results obtained from Table 4 are very good. For target 1, which was the imagined movement of the right hand, the interface generated over 74% of this feature vector for all participants. This means that the interface correctly predicted target 1 in more than 74% of cases and displayed an arrow pointing to the right on the monitor screen. For target 2, which was the imagined movement of the left hand, the results are slightly lower. The interface correctly generated target 2 in over 70% of this feature vector for all participants.

### 3.2. EEG Data Analysis Using Machine Learning

In recent years, there has been a dynamic development of machine learning techniques applied to the analysis of electroencephalographic (EEG) brain signals. Traditional machine learning approaches, such as Naïve Bayes, linear regression (LR), random forests (RF), and support vector machines (SVM), have been widely used for EEG signal classification, but they encountered significant limitations. These limitations were related to manual feature selection and poor generalization ability across different tasks and research populations. Feature extraction was essential before feeding data into actual machine learning classification algorithms. It allowed for the extraction of relevant information from raw EEG signal data, which could be more efficiently utilized by any machine learning model. Typical feature extraction methods from raw EEG data include frequency and spatial filtering, as well as time- and frequency-domain analyses. Depending on the type of data and applied technique, features such as signal power in specific frequency bands or spectral parameters can be obtained. Statistical indicators, including signal variance, autoregressive coefficients, and entropy measures, are also commonly used to capture characteristic brain activity patterns. After obtaining the desired features, a traditional machine learning classification algorithm is implemented to train and test the system [3,35].

In response to these numerous challenges, modern deep learning (DL) techniques have emerged as powerful tools for EEG signal analysis. Traditional approaches, such as convolutional neural networks (CNNs) and recurrent neural networks (RNNs), have achieved significant success in classifying EEG patterns associated with anxiety. CNNs are particularly effective in capturing spatial dependencies in EEG data, while RNNs are suited for modeling temporal sequences. However, CNNs may struggle with sequential dependencies over long time intervals, and RNNs often suffer from vanishing gradient problems, limiting their ability to model long-term dependencies.

Recently, transformer-based models have been introduced to EEG research. Unlike RNNs, transformers use self-attention mechanisms to model global temporal dependencies without the limitations of gradient degradation, making them highly suitable for detecting complex and subtle patterns in EEG signals. Studies such as Pfeffer et al. (2024) [35] have demonstrated that transformer architectures can outperform CNNs and RNNs in EEG-based brain–computer interface (BCI) tasks, offering higher classification accuracy and better generalization. However, transformers generally require large amounts of training data, which may limit their application in small-sample studies such as ours.

To address the issue of data scarcity, generative models have been increasingly used to augment EEG datasets. In our study, we explored the application of the VAE-D2GAN model. Traditional Generative Adversarial Networks (GANs) often struggle with issues such as mode collapse and unstable training, while Variational Autoencoders (VAEs) may produce blurred samples that fail to capture fine details of EEG signals. The VAE-D2GAN model combines the advantages of both approaches by integrating variational inference with a dual discriminator GAN architecture, aiming to generate more diverse and high-fidelity synthetic EEG samples.

According to Bao et al. (2021) [36], VAE-D2GAN provides improved sample diversity and stability compared to conventional GANs and VAEs when generating emotional EEG data. This is particularly crucial for anxiety detection, where emotional brain states can be heterogeneous and subtle. Nevertheless, synthetic data generated by VAE-D2GAN must be carefully validated to ensure that no artificial biases are introduced, which could otherwise compromise the reliability of machine learning models trained on such data. Overall, by integrating transformer-based models for classification and VAE-D2GAN for data augmentation, there is a promising pathway toward improving EEG-based anxiety detection. However, the limitations related to large data requirements and validation of synthetic datasets must be addressed in future work.

Concurrently, intensive research is ongoing into the application of real-time EEG systems for anxiety detection. Modern portable EEG systems enable monitoring of the user’s emotional state outside of the laboratory, opening new possibilities in the treatment of anxiety disorders. An example of this is SCORE-AI, a system that uses advanced machine learning algorithms for the automatic interpretation of EEG studies in anxiety diagnostics, achieving accuracy comparable to expert analysis [23]. EEG biofeedback systems, based on machine learning methods, are also increasingly used in therapy, allowing patients to consciously regulate brain activity in order to reduce anxiety symptoms [20].

Current research indicates the need to strengthen and expand the potential for integrating EEG with multimodal neuroimaging data, such as functional magnetic resonance imaging (fMRI) and magnetoencephalography (MEG). This could lead to even more accurate diagnostic methods [10]. However, the following key challenge remains: ensuring the interpretability of machine learning models in EEG analysis and adapting them to different patient groups and clinical environments.

### 3.3. Examples of AI Applications in Medicine

Artificial intelligence (AI) plays a crucial role in the rapidly evolving healthcare IT sector by enhancing the efficiency and effectiveness of medical professionals. AI is driving transformation in neurology, particularly in the diagnosis and treatment of anxiety disorders. Machine learning (ML) algorithms enable the analysis of large datasets to identify previously undiscovered patterns and relationships, which is essential for integrating ML into diagnostic and prognostic neurology and for designing future therapies for individuals with various mental disorders.

In neurology, ML algorithms are used to analyze EEG signals to detect patterns associated with anxiety, epilepsy, and neurodegenerative disorders. One example is the SCORE-AI system, which autonomously interprets routine EEG examinations with performance comparable to human experts, achieving AUC values between 0.89 and 0.96 in detecting EEG abnormalities. SCORE-AI not only distinguishes normal from abnormal EEGs but also classifies abnormalities into clinically relevant categories—epileptiform-focal, epileptiform-generalized, nonepileptiform-focal, and nonepileptiform-diffuse. Such tools can enhance diagnostics and patient care, especially in regions with limited access to specialists, by providing accurate, automated interpretations. Additionally, SCORE-AI has the potential to improve efficiency and consistency in specialized epilepsy treatment centers by reducing expert workload, allowing them to focus more on complex cases. Unlike previous AI models, which primarily detected epileptiform activity, SCORE-AI offers a more nuanced classification of EEG abnormalities, supporting clinical decision-making and guiding treatment strategies [23].

Another key area is the development of intelligent brain–computer interfaces (BCIs), which allow control of external devices solely through brain activity. BCIs explore the relationship between human behavioral rules and computer programs simulating these behaviors. Recording these behaviors as distinctive brain activity patterns enables their future use in AI technologies. BCIs have practical applications in daily activities, communication, entertainment, rehabilitation, and prosthetics. They provide individuals with disabilities the ability to control assistive devices and allow those unable to communicate through conventional means to write using only neural activity. Ongoing research focuses on aiding individuals with disabilities through BCI technology, particularly for patients with conditions such as multiple sclerosis or Guillain–Barré syndrome. Studies (e.g., Jackson and Mappus, 2010 [19]) demonstrated that BCIs could enable motor-impaired patients to communicate desires or express opinions by moving a cursor to indicate “yes” or “no.”

## 4. Discussion

Despite the rapid development of artificial intelligence methods and their growing role in enhancing brain–computer interfaces (BCIs), a significant proportion of users still struggle to effectively operate these systems. It is estimated that 15% to 30% of participants in BCI studies experience what is termed “BCI illiteracy”, presenting a major challenge to the widespread adoption of this technology.

To address this issue, various strategies are employed, including advanced training for both users and classifiers, the development of more intuitive interaction scenarios, improvements in EEG signal processing algorithms, and the elimination of technical faults. However, despite these efforts, only a subset of users show improvement, while full control over the interface may remain unattainable for others. Future research should focus on improving BCI accessibility, particularly addressing BCI illiteracy through adaptive algorithms and personalized training protocols. Specifically, future work should prioritize the development of adaptive machine learning models capable of real-time adjustment to individual neural profiles, leveraging techniques such as transfer learning and online learning to support users with initially low BCI performance.

The effectiveness of BCI usage is influenced by numerous factors, including individual differences in brain microstructure that can affect the quality of recorded EEG signals. In some cases, brain activity may be too weak to detect or may be masked by more active neighboring neurons, resulting in a low signal-to-noise ratio.

During the study, it was also observed that certain physiological characteristics of participants could pose an additional obstacle to obtaining precise measurements. One particularly problematic factor was the presence of very dense hair, which made it difficult for the electrodes to properly adhere to the scalp. A notable example was one of the experiment participants (number 1), whose thick hair resulted in imprecise electrode placement, potentially leading to the occurrence of technical and physiological artifacts and, consequently, a deterioration in the quality of the recorded EEG signals.

Stress can impair concentration and the ability to absorb new information, which is crucial in tasks requiring focus and learning, such as the training session before the EEG. The participant with the lowest results might have had difficulty fully engaging in the study, as their attention could have been partially occupied by the upcoming exam. Even though they described the test as interesting, subconscious stress and tension might have limited their ability to relax, ultimately affecting their final results. Our observations are consistent with previous research highlighting the role of training and situational stress in BCI effectiveness (Nijboer et al., 2008) but were first observed in the context of natural academic stress [37].

This finding highlights the crucial importance of training sessions, especially when working with individuals suffering from anxiety disorders. Based on interviews with the participants, it became evident that structured training sessions help individuals gradually adapt to the test conditions, reducing stress and improving overall performance. Therefore, incorporating preparatory sessions is essential when conducting EEG studies with anxious individuals, as they provide a necessary adjustment period that can lead to more reliable results and better engagement in the study.

It is important to emphasize that this research is a pilot study involving only three participants. This small sample size precludes the use of inferential statistical methods, such as confidence intervals or hypothesis testing. As a result, the classification accuracy outcomes presented should be interpreted with caution due to the high risk of overfitting and limited statistical robustness. We acknowledge that the findings serve primarily to assess methodological feasibility and identify preliminary trends, which require confirmation in follow-up studies with larger, more diverse populations.

Additional barriers include muscle artifacts, misinterpretation of BCI operation instructions, and procedural issues such as improper electrode placement or software errors. While many of these factors can be mitigated, individual neurophysiological differences may continue to pose significant challenges. Future advancements must focus on refining algorithms, personalizing BCI systems, and developing alternative brain activity imaging techniques to enhance the accessibility and effectiveness of BCI technology. Our findings primarily reflect EEG responses to subclinical, situationally induced anxiety, which may differ from the patterns observed in patients with chronic anxiety disorders. This distinction should be considered when interpreting the generalizability of the machine learning models.

## 5. Conclusions

The integration of EEG and machine learning opens new avenues for exploring the relationship between brain structures and emotional states. This combination allows the identification of key neurobiological features involved in emotional regulation and may, in the future, enable more precise predictions of anxiety onset—facilitating early interventions and more effective treatments.

Our experimental findings confirm that repeated training sessions improve EEG signal classification accuracy, highlighting the importance of adaptive and personalized approaches. These improvements are essential for enhancing brain–computer interface (BCI) systems, which can support the development of dynamic, self-learning tools for anxiety detection and intervention.

While this pilot study demonstrates promising directions, future research should address long-term clinical efficacy, patient acceptance, and ethical considerations such as data privacy and security.

## Figures and Tables

**Table 1:** Results of k-fold cross-validation.

Survey Participant	k-Fold Cross-Validation [%]								Sigma [%]
	Step 1	Step 2	Step 3	Step 4	Step 5	Step 6	Step 7	Average	
1	63.928571	79.642857	79.642857	63.214286	82.142857	80.357143	68.214286	73.87755	7.760135
2	70	80.357143	56.428571	66.785714	79.285714	83.571429	44.285714	68.673469	13.207685
3	95	86.785714	97.5	99.285714	97.5	78.571429	86.428571	91.581633	7.1705

**Table 2:** Results of k-fold cross-validation.

Survey Participant	k-Fold Cross-Validation [%]								Sigma [%]
	Step 1	Step 2	Step 3	Step 4	Step 5	Step 6	Step 7	Average	
1	53.714286	52.857143	39.357143	47.232857	60.589429	15.143523	55.231425	46.303687	13.135478
2	50.714286	37.857143	34.285714	35.357143	68.214286	17.142857	42.142857	40.816327	14.599506
3	44.285714	63.571429	47.5	52.857143	48.928571	72.5	52.857143	54.642857	9.226657

**Table 3:** The results of the confusion matrix.

Survey Participant	Cls vs. Cls [%]			
	Input 1		Input 2	
	1	2	1	2
1	78.6	21.4	30.8	69.2
2	70.7	29.3	33.4	66.6
3	92	8	8.9	91.1

**Table 4:** Results of confusion matrix for learning task.

Survey Participant	Cls vs. Cls [%]			
	Input 1		Input 2	
	1	2	1	2
1	79	21	26.7	73.3
2	74.5	25.5	28.7	71.3
3	92.3	7.7	8.1	91.9

## Data Availability

The original contributions presented in this study are included in the article. Further inquiries can be directed to the corresponding author. The data are not publicly available due to sensitive information contained.
